# The impact of Arctic warming on increased rainfall

**DOI:** 10.1038/s41598-018-34450-3

**Published:** 2018-10-30

**Authors:** R. Bintanja

**Affiliations:** 1 Royal Netherlands Meteorological Institute (KNMI), Utrechtseweg 297, 3731 GA De Bilt, The Netherlands; 2 Energy and Sustainability Research Institute Groningen (ESRIG), University of Groningen, Nijenborgh 6/7, 9747 AG Groningen, The Netherlands

## Abstract

The Arctic region is warming two to three times faster than the global mean, intensifying the hydrological cycle in the high north. Both enhanced regional evaporation and poleward moisture transport contribute to a 50–60% increase in Arctic precipitation over the 21^st^ century. The additional precipitation is diagnosed to fall primarily as rain, but the physical and dynamical constraints governing the transition to a rain-dominated Arctic are unknown. Here we use actual precipitation, snowfall, rainfall output of 37 global climate models in standardised 21^st^-century simulations to demonstrate that, on average, the main contributor to additional Arctic (70–90°N) rainfall is local warming (~70%), whereas non-local (thermo)dynamical processes associated with precipitation changes contribute only 30%. Surprisingly, the effect of local warming peaks in the frigid high Arctic, where modest summer temperature changes exert a much larger effect on rainfall changes than strong wintertime warming. This counterintuitive seasonality exhibits steep geographical gradients, however, governed by non-linear changes in the temperature-dependent snowfall fraction, thereby obscuring regional-scale attribution of enhanced Arctic rainfall to climate warming. Detailed knowledge of the underlying causes behind Arctic snow/rainfall changes will contribute to more accurate assessments of the (possibly irreversible) impacts on hydrology/run-off, permafrost thawing, ecosystems, sea ice retreat, and glacier melt.

## Introduction

Changes in Arctic rainfall are dominated by two distinct processes. Firstly, changes in total precipitation, which in turn can be attributed to atmospheric moisture transports (mainly from lower latitudes) converging over the Arctic (through warmer air being able to hold more moisture and/or by changing atmospheric dynamics, including variability in the jet stream associated with changes in the North Atlantic Oscillation)^1–3^, to sea-ice-retreat induced surface evaporation changes^4^, and to (uncertain) microphysical processes that transform atmospheric moisture into clouds and precipitation^3^. Secondly, changes in local temperature (of the lowest atmospheric layers), which govern whether or not the precipitation that starts off as snow will melt into rain on its way to the surface. Changes in precipitation and their underlying causes can be characterized as ‘remote’ because they depend primarily on non-local climate mechanisms, whereas changes in local temperature directly determine the transformation of snow to rain. Since the scale, magnitude and duration of various impacts of Arctic precipitation trends depend crucially on whether the additional precipitation will fall as snow or rain^5^, it is imperative to accurately determine the underlying causes behind the steep increase in projected Arctic rainfall, including its dependence on local warming.

The relation between changes in Arctic rainfall and both temperature and precipitation is surprisingly complex, however. Obviously, higher temperatures lead to a lower snowfall fraction and thus more rain, but the rate of increase depends crucially on the ambient atmospheric temperature. In very cold conditions, temperature increases will hardly affect the snowfall fraction, which will stay close to 1. Similarly, in temperatures sufficiently far above 0 °C where snowfall fractions are close to zero, warming also will not lead to substantial increases in liquid precipitation. The largest impact on the snowfall fraction, and thus on temperature-induced rainfall changes, will occur in the temperature range close to 0 °C (ref.^6^) (see Suppementary Information). In the Arctic, the largest temperature increases are projected to occur during the cold winters, and to a much lesser extent in the already fairly mild summers^5,7^. Moreover, projected increases in Arctic precipitation exhibit a strong seasonal signature, peaking in late autumn and winter^4^. Hence, the impact of local warming on rainfall changes will strongly and non-linearly depend on the background climate (hence geographical location), and on the season.

To what extent impacts of increased Arctic rainfall can be attributed to local warming or to remote (thermo)dynamical processes is key to fully understand the causes, magnitude and irreversability of the various impacts and to develop appropriate adaptation strategies. Potential impacts include the snow-covered surface reflectivity (rainfall lowers the albedo whereas snowfall increases the albedo^3,8^), the melting of snow and sea ice through heat transfer by rainfall penetrating into the snow pack^9^, hydrological changes^10–12^ (e.g. by changing the seasonality of river runoff and Arctic Ocean vertical mixing) and the melting of permafrost^13^ (and consequent release of greenhouse gases^14^), all of which occur more efficiently during rainfall. Moreover, more rain will lead to ecological changes associated with icing (limiting food availability for animals^15^), and with run-off affecting marine biochemistry^16^ and Arctic ecosytems^17,18^, to enhanced glacier melt^19^, and to economic impacts through more frequent icing conditions^20^ and to permafrost melting impacts on infrastructures and indigenous people^21^.

## Attribution of Rainfall Changes

**Figure 1:**
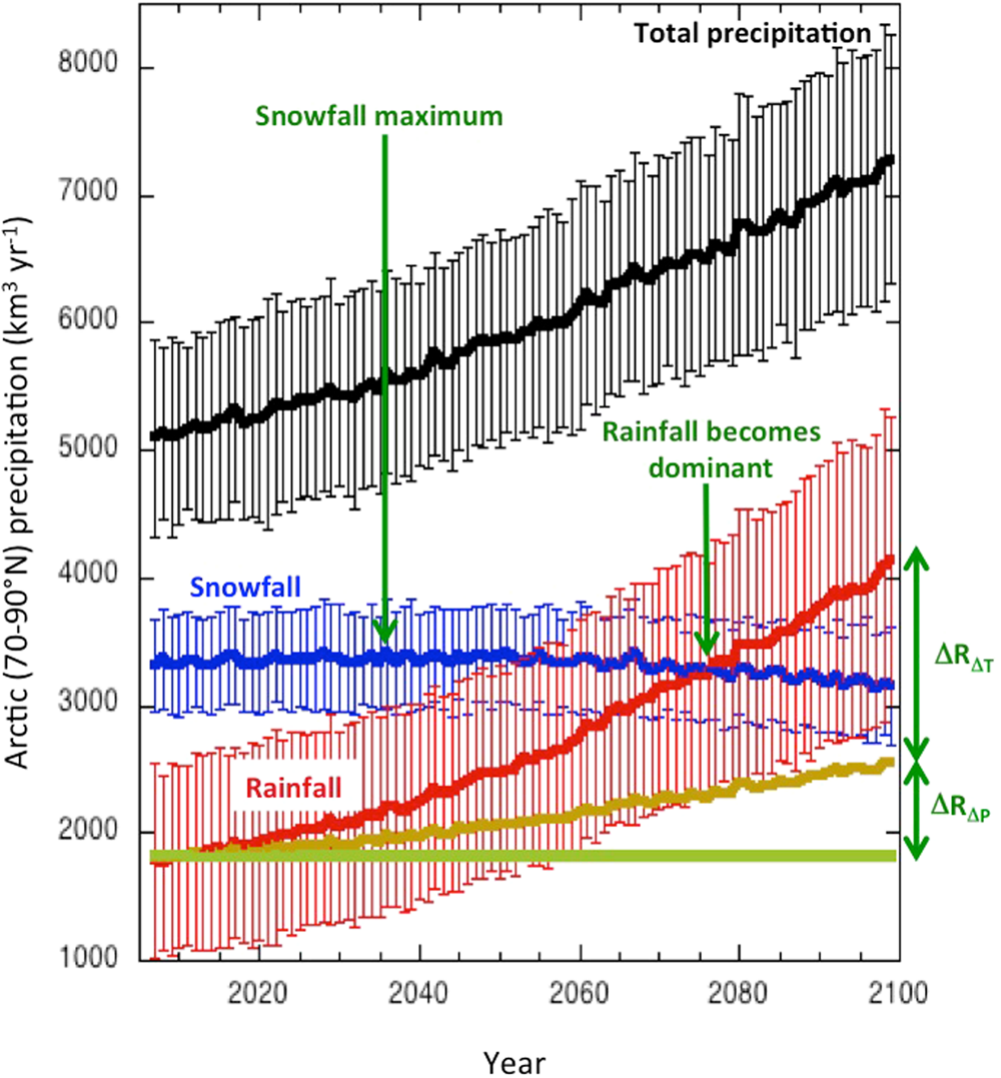
Projected climate model mean (37 models), Arctic mean (70–90°N) and annual mean changes in total precipation, snowfall and rainfall. The contributions of temperature and precipitation changes to Arctic rainfall are indicated by ΔR_ΔT_ and ΔR_ΔP_, respectively (see Supplementary Information). Error bars represent the multi-model standard deviation and indicate model uncertainty.

Here we apply 37 state-of-the-art global climate models in standardised scenario simulations (RCP8.5, 2006–2100)^7^ to quantify future changes in Arctic rainfall and identify/evaluate the underlying processes. We use actual climate model output of surface air temperature, total precipitation and snowfall/rainfall. Arctic mean (70–90°N) precipitation is projected to strongly increase over the next century (Fig. 1), with the increase accelerating towards later stages of the century^4^. The increase in Arctic rainfall even outpaces that in total precipitation, aided by the fact that, on average, vigorous Arctic warming causes solid precipitation to melt into rain^5^. Virtually all precipitation starts as snow, especially in the polar regions, but thawing temperatures in atmospheric layers between the surface and the cloud base cause the snow to melt and transform into rain. This melt process, which is essentially driven by local temperature trends, is the main contributor to the increase in Arctic rainfall (~70%). In the (hypothetical) absence of this process, Arctic rainfall would still increase simply because the extra precipitation would transform into rain at the same rate (or snow fraction, defined as the fraction of snowfall to total precipitation) as today (30%). Increases in total precipitation are to a considerable extent associated with changes in (remote) climate variables through changes in moisture transport, sea-ice related surface evaporation and cloud microphysical processes^2,4^. In any case, the sharp increase in snowfall being transformed into rain causes Arctic mean snowfall to hardly increase in the near-future and even decline after the year 2035. As a result, the combined models project that rainfall will become the dominant form of Arctic precipitation around the year 2080 (in the RCP8.5 scenario).

**Figure 2:**
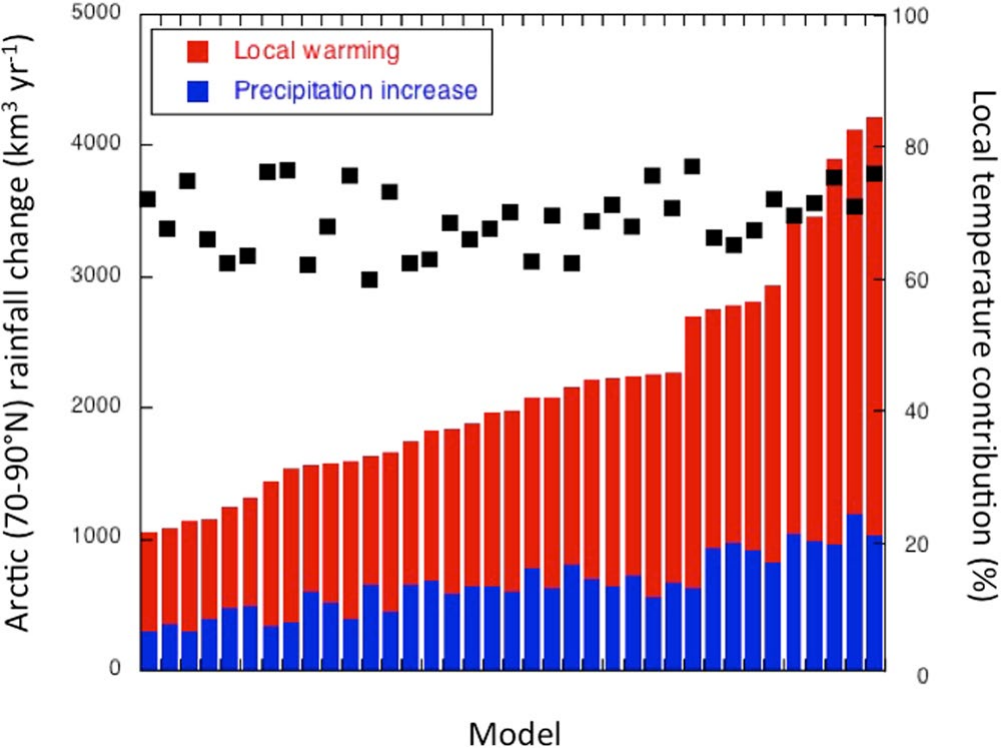
Projected Arctic mean (70–90°N) and annual mean changes in rainfall, including the contributions of changes in local temperature and precipitation, for each climate model. Black squares indicate the percentage by which local warming determines rainfall changes.

While all 37 climate models agree that Arctic rainfall will increase over the next century, the magnitude of the simulated changes is much less robust among models (Fig. 2). The increase in Arctic rainfall varies by a factor of 4 between the most extreme models, but in all models local temperature changes govern increases in annually averaged Arctic rainfall. In fact, the relative contribution of local warming on changes in rainfall is remarkably constant among the various models, varying between 60 and 80%. This means that the temperature-dependent transition from snow to rain is a shared feature in the intermodel sense, despite the strong intermodel variation in Arctic warming rates (models project a 4 to 13 °C Arctic mean and annual mean 21^st^-century warming under RCP8.5 forcing^2,4^). Apparently the increases in total precipitation and (local warming induced) absolute increases in rainfall are such that the fractional contribution of local warming induced rainfall is largely model independent and therefore a robust feature of Arctic climate change. However, considering annual and Arctic means averages out strong spatial and seasonal variations in rainfall changes and in the associated climate processes.

## Spatial and Seasonal Variations

**Figure 3:**
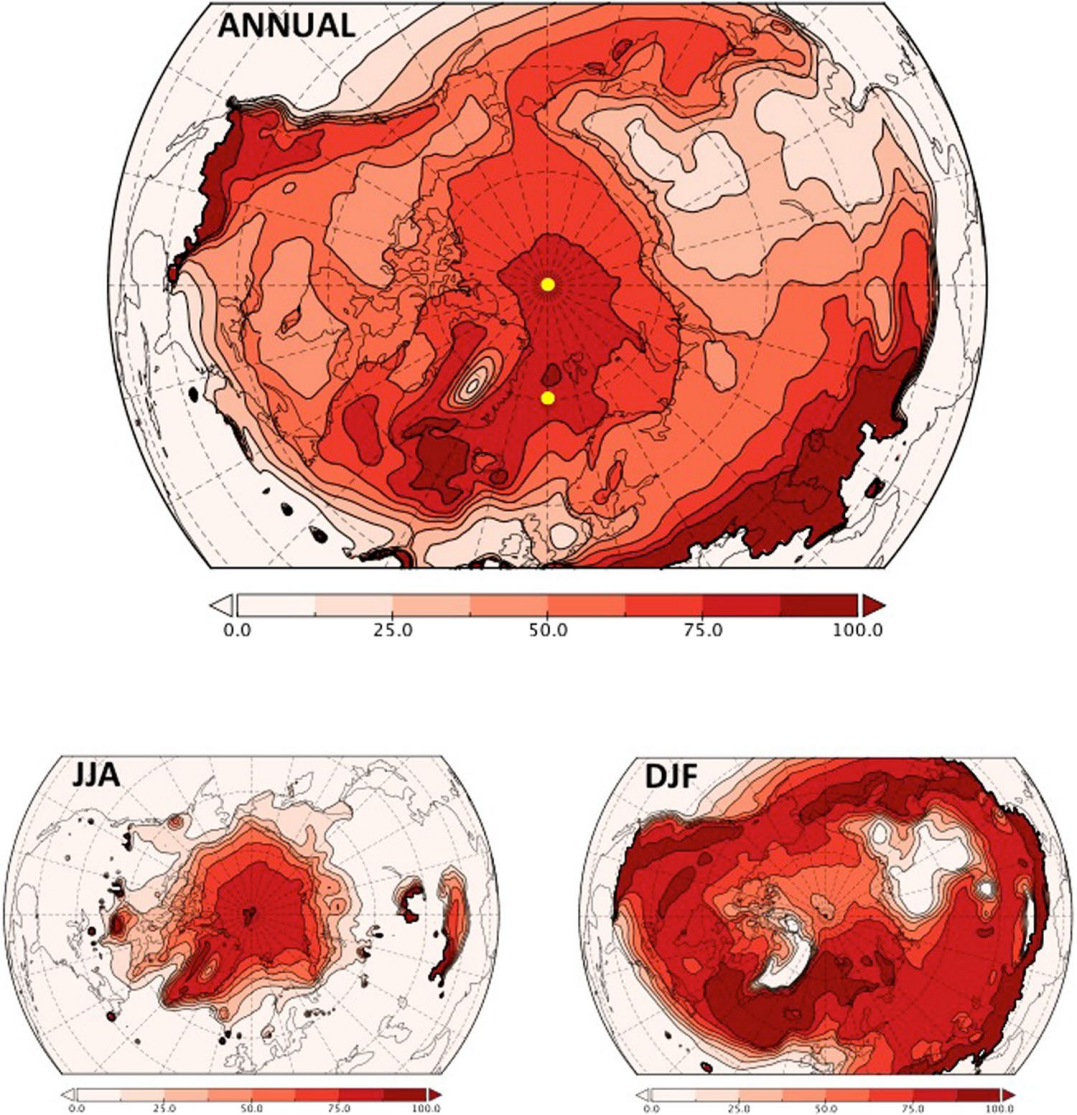
Projected Arctic rainfall changes attributed to changes in local temperature, for annual mean (top), summer (JJA, June-July-August; bottom left) and winter (DJF, December-January-February; bottom right). 100% percent means that the increase in rainfall can be totally attributed to local warming. Yellow dots in the top panel show the locations (one on the North Pole and one in the North Atlantic) used in Fig. 4.

The effect of local warming on rainfall changes exhibits a complex geographical distribution, with peak values over the Arctic Ocean and North Atlantic, comparatively low values over the subarctic continents and again higher values in the mild southern regions (Fig. 3a). The fact that the local warming effect peaks in the high Arctic Ocean is a unexpected result, as one would instinctively not assume this factor to dominate in the coldest regions of the Arctic where liquid precipitation is relatively scarce to begin with. However, it is the region where projected temperature changes are largest (a phenomenon coined Arctic amplification)^2,4^, potentially amplifying the temperature effect on rainfall changes. Interestingly, the maximum values over the central Arctic Ocean can be attributed largely to summer conditions (Fig. 3b), during which changes in snowfall fraction are maximum in the central Arctic where current summer temperatures are around the freezing point. In the subarctic regions, present-day temperatures are too mild (and future warming too modest) to invoke large increases in snowfall fraction. In winter (Fig. 3c), the situation is largely reversed, with minimum values of the temperature effect on rainfall over the high Arctic and the subarctic continents (especially Siberia and Greenland where current winter temperatures are extremely low). This geographical pattern closely resembles that of the trends in snowfall fraction^5^, indicating that these regions are currently too cold to allow local warming to cause snowfall to melt, even considering the projected strong winter warming rates over the central Arctic.

**Figure 4:**
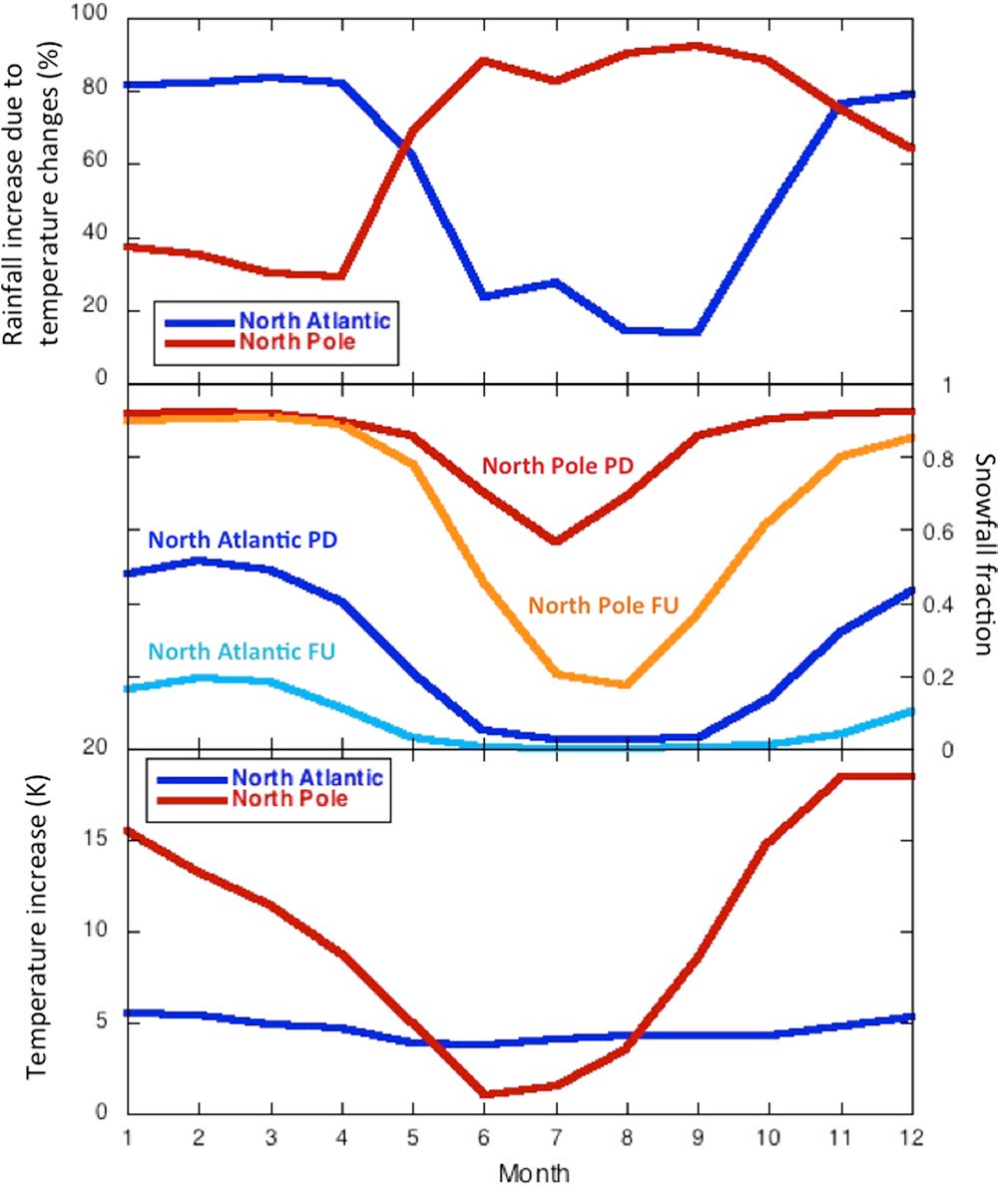
Seasonal variation in warming-induced rainfall increase (top), present-day and future snowfall fractions (middle), and surface warming (bottom), for two locations: one on the North Pole (90°N; red/orange) and one in the North Atlantic (75°N, 0°W; blue/light blue). Both locations are indicated in Fig. 3 (top panel) as yellow dots. PD refers to present-day (2006–2015 mean) while FU refers to future (2091–2100 mean).

This finding suggests that a strongly non-linear interaction between background climate, local warming and precipitation changes causes the local warming effect on rainfall to exhibit steep geographical gradients in its seasonal cycle (Fig. 4), see Supplementary Information. Two locations with roughly the same annual value in local temperature induced rainfall (ΔR_ΔT_), one in the North Atlantic and the other on the North Pole, exhibit totally opposing seasonal cycles (Fig. 4a). Over the central Arctic Ocean ΔR_ΔT_ peaks in summer, whereas over the North Atlantic ΔR_ΔT_ reaches maximum values during the frigid winter months. This reversal is decidedly linked to seasonal changes in the temperature-dependent snowfall fraction: ambient temperatures should be around zero to allow local warming to have an appreciable effect on the snowfall fraction (see Supplementary Information). In too cold (central Arctic winter) or too mild (subarctic summer) conditions, temperature changes do not lead to substantial changes in the snowfall fraction (Fig. 4b), even for projected winter warming rates as high as about 20 °C in de central Arctic (Fig. 4c). Therefore, paradoxically, the projected extreme winter warming in the central Arctic Ocean will only lead to minute effects on rainfall, whereas quite moderate summer warming rates will cause a strong increase in rainfall. This stems from the non-linear dependence of snowfall fraction on temperature (change)^6^, and results in steep spatial gradients in the magnitude and seasonality through which projected future changes in Arctic rainfall can be linked to local warming.

This links to the issue whether changes in Arctic rainfall – and thereby snowfall^22^ – can be attributed primarily to local temperature increase (melting snowfall into rain) or to more generic changes in total precipitation (which are governed by larger-scale processes such as poleward moisture transport, tropospheric warming, synoptic activity (transient cyclones), jet stream dynamics, surface evaporation and cloud formation processes^1–3^). Climate models are consistent in their projections of the relative contribution of local temperature on Arctic average rainfall changes, but superimposed on these are huge spatial and seasonal variations. During winter over the Arctic Ocean and the adjacent continents, changes in rainfall are effectively decoupled from local warming owing to minute changes in snowfall fraction in these very cold climates. Hence, in such deep-freeze conditions, additional rainfall can be attributed mainly to large-scale precipitation changes, and local warming rates are not a good predictor of rainfall changes.

## Conclusions

The intuitive notion that strong Arctic warming always leads to considerable increases in rainfall is inaccurate, even though on average (Arctic and annual mean) local warming does explain the major part (~70%) of the increase in rainfall. Our findings also link to the uncertainty in future projections of Arctic rainfall and snowfall: local, seasonal and temporal variability in precipitation potentially exhibits a different – and likely larger (due mainly to uncertain cloud-related processes^3^) – uncertainty than those in local temperature^23^. Addressing the magnitude, extent and potential irreversability of the various impacts of projected strong increases in Arctic rainfall (such as hydrology/run-off, permafrost thawing, ecosystem shifts, sea ice retreat, and glacier melt) will benefit from improved insights into the underlying causes, in particular the spatial/seasonal dependence of the attribution of rainfall to local warming, the associated accuracy in the projections and the effect on seasonally varying snowfall rates.

## Methods

In all analyses we used the Coupled Model Intercomparison Project, phase 5 (CMIP5) state-of-the-art global climate models^7^, which were driven by standardised forcing scenarios for the period 2006–2100. Here we use the strong (RCP8.5) forcing scenario, for which the combined greenhouse, aerosol and other radiative forcings in the year 2100 totals 8.5 W m^−2^ (ref.^7^). We use all models for which the coverage of surface air temperature, precipitation and snowfall output was complete and without obvious errors (other than that no selection of models was made); one ensemble member per model (the first) was used. 21^st^-century trends in Arctic temperature, precipitation and its solid and liquid components are defined as differences between the means over the periods 2091–2100 and 2006–2015 (refs^4,5^), with all uncertainties throughout the paper being evaluated as the standard deviations of the model ensemble. Note that annual means of thermal contribution fractions (Figs 2 and 3) were evaluated by first calculating absolute (monthly) values, then integrate over one year, and finally take the ratio of the thermal contribution and total rainfall.

## Electronic supplementary material

Supplementary Information

## Data Availability

All climate model data used in this paper are available through the CMIP data portals.
