# Supplementary material for: The impact of Arctic warming on increased rainfall
Source: Sci Rep. 2018 Oct 30;8:16001. doi: 10.1038/s41598-018-34450-3 (PMC6207739; doi:10.1038/s41598-018-34450-3)
Supplement: Supplementary file 1 — Supplementary Information [file 41598_2018_34450_MOESM1_ESM.docx]

**Supplementary Information**

The impact of Arctic warming on increased rainfall – R. Bintanja

To elucidate the temperature-dependency of changes in rainfall in the climate model output presented in the main paper, we put forward a more theoretical framework in which we analyse changes in total precipitation (*P*) as well as its components snowfall (solid precipitation, *S*) and rainfall (liquid precipitation, *R*) over the 21^st^ century. Changes in *P*, *S* and *R* can be written as:

$$\Delta P = P_{1} - P_{0} (1)$$

$$\Delta S = S_{1} - S_{0} (2)$$

$$\Delta R = R_{1} - R_{0} (3)$$

in which subscript 0 refers to the average over the period 2006-2015 (present-day), while subscript 1 refers to the average over the period 2091-2100 (future). Since *P* = *S* + *R*, we can express Δ*P* as:

$$\Delta P = \Delta S + \Delta R (4)$$

The snowfall fraction *f* is commonly defined as the fraction of solid to total precipitation:

$$f = \frac{S}{P} (5)$$

and 21^st^-century changes in *f* can be expressed as:

$$\Delta f = f_{1} - f_{0} (6)$$

Using various snow/precipitation observational datasets, Krasting et al. (2014) were able to empirically link the snowfall fraction to atmospheric surface air temperature *T* (monthly mean values, in °C) according to:

$$f(T) = \frac{1}{1 + a\cdot b^{T}} (7)$$

in which they found that values of $a$ = 1.61 and $b$ = 1.35 best represented the observations. The slope of this relation is always negative (Figure S1), indicating that warming always leads to lower values in *f*, and thus to an increased fraction of total precipitation falling in liquid form. However, the decline in *f* with increasing temperatures is slim in either very cold or very mild climates: in cold climates for which *f* ≈ 1 (virtually all precipitation is solid), warming will not lead to appreciable reductions in *f*; the same holds for very mild climates (almost all precipitation is liquid to begin with). It is the intermediate temperature range around the freezing point of water for which changes in temperature lead to the largest changes in *f*. This non-linearity explains most of the features related to the seasonal and geographical variations in snow/rainfall changes in the Arctic and subarctic regions as depicted by the climate model output (Figures 1-4).

Combining expressions (1) through (6) and some algebraic manipulation leads to expressions for Δ*S* and Δ*R* in terms of temperature and total precipitation changes:

$$\Delta S = P_{1}\Delta f + f_{0} \Delta P (8)$$

$$\Delta R = {-P}_{1}\Delta f + {(1 - f}_{0})\Delta P \equiv\Delta R_{\Delta T} + \Delta R_{\Delta P} (9)$$

where $\Delta R_{\Delta T} =$ ${-P}_{1} \Delta f$is defined as the component of changes in rainfall that can be attributed to temperature changes (the part of snowfall that is transformed into rainfall by above-zero temperatures between the surface and cloud base), whereas $\Delta R_{\Delta P} = {(1 - f}_{0})\Delta P$ represents the component related to changes in total precipitation. Note that under climate warming, $\Delta f$ is negative by virtue of (7), meaning that both terms on the right-hand side of (9) are positive and thus contribute to rainfall increases. The temperature-dependency of $\Delta R_{\Delta T}$ is governed by $\Delta f$, which through (7) will peak in the temperature range around *T* = 0 °C. Note also that the precipitation-induced rainfall change ($\Delta R_{\Delta P}$) is very small in cold climates where $f_{1}$ ≈ 1, simply because then virtually any change in precipitation will be in the solid form.

*Figure S1. Empirical relation between snowfall fraction on surface air temperature (Krasting et al., 2014) based on various precipitation/snowfall datasets according to (7) with a = 1.61 and b = 1.35.*

Using (9), the relative contribution of local temperature changes to the total increase in rainfall ($\xi$) can be expressed as:

$$\xi\equiv\frac{\Delta R_{\Delta T}}{\Delta R} = \frac{\Delta f}{\begin{aligned} \Delta f + (1 - f_{0})({P_{0}}/{P_{1}- 1)} \\ \end{aligned}} (10)$$

Clearly, $\xi$ depends non-linearly on the background climate state ($f_{0}$), on climate warming ($\Delta f$) and the change in total precipitation (${P_{0}}/{P_{1}}$). Note that in a warming Arctic, all terms in (10) are generally negative since $P_{1}$ > $P_{0}$ virtually everywhere. Also note that the various variables are not independent of each other, since for instance if $f_{1}$ ≈ 1 (in very cold climates) then $\Delta f$ will be very small for reasonable warming rates by virtue of (7). Also precipitation rates will be comparatively small in cold climates, so ${P_{0}}/{P_{1}} \to1$. From this expression it can be deduced that in very cold climates $\Delta f \to0$ (and $f_{0} \to1$), meaning that the temperature-caused rainfall increases will be minute. Hence, warming in a very cold climate does not lead to substantial increases in rainfall. In the more moderate Arctic or subarctic climates (*T* ≈ 0 °C), $\Delta f$ is maximum according to the climate models (Bintanja and Andry, 2017). If these climate states will experience a moderate increase in total precipitation (which according to the climate models seems to be the case) then $\xi$ can reach values close to 1. This suggests that in such regions/seasons nearly 100% of the additional rainfall can be attributed to local warming, even though near-surface warming is much less pronounced than in the high Arctic.

In any case, combining relation (10) with the empirical relation (7) explains why $\xi$ exhibits such strong seasonal and geographical variations. For instance, the associated non-linearities cause increases in rainfall in very cold climate regimes (e.g. subarctic continental winters) to be largely decoupled from local climate warming, which confirms the climate model results (Figure 3b).

One should note that the emperical relation (7) is strictly valid only for present-day conditions over the Northerns Hemisphere continents. This means that its application in the high Arctic and in future climates will introduce additional uncertainty in the above interpretations (note that the results in the main paper are based solely on actual climate model output), since weaker thermal and specific humidity inversions will change the relation between surface temperature and the vertical temperature structure.

**References**

Bintanja, R. & Andry. O. Towards a rain-dominated Arctic. *Nature Climate Change*, **7**, 263–267 (2017).

Krasting, J., Broccoli, A., Dixon, K. & Lanzante, J. Future changes in northern hemisphere snowfall. *J. Clim.* **26**, 7813–7828 (2013).
